# Component Separation: A Case Report of Hybrid and Synthetic Absorbable Mesh Use for Complex Large Ventral Hernia Reparation

**DOI:** 10.7759/cureus.36347

**Published:** 2023-03-19

**Authors:** Harold Bafitis, Vania Arboleda, Isabel Bernal

**Affiliations:** 1 Surgery, Kiran C. Patel College of Osteopathic Medicine, Nova Southeastern University, Fort Lauderdale, USA; 2 Medicine, Kiran C. Patel College of Osteopathic Medicine, Nova Southeastern University, Fort Lauderdale, USA

**Keywords:** synthetic mesh, hybrid mesh, double mesh technique, abdomen ventral hernia, component separation technique

## Abstract

Ventral abdominal hernias are a common abdominal wall defect in the United States. We present a 50-year-old Caucasian male with a large (>18 cm) abdominal wall defect. An extensive complex abdominal wall reconstruction with advanced bilateral fascial flaps/component separation and repair of the abdominal wall defect was planned to restore the appropriate abdominal wall anatomic contour. The use of double mesh in large abdominal wall defects is still a relatively new documented technique. Only two case series detail the same technique used on this patient, with no articles on using a hybrid mesh with a synthetic absorbable mesh. This case uses an underlay and onlay mesh technique, with a hybrid mesh, Tela Biologics (Malvern, PA, USA), under the muscle, in this case, intraperitoneal bridging the gap. The anterior rectus sheath was reinforced with intercepted 0-Ethibond sutures (Ethicon/J&J, Bridgewater, NJ, USA) and then reinforced with a synthetic absorbable mesh (Phasix^TM^, Becton Dickinson, Franklin Lakes, NJ). The outcome with this patient shows more research should be conducted on considering long-term results with the types of mesh and the question of whether there are additional benefits when using two different types of mesh and their placement in the sandwich technique.

## Introduction

An abdominal wall hernia involves a protrusion of intra-abdominal tissue through a fascial defect in the abdominal wall. Inguinal hernias are the most common abdominal type, accounting for approximately 75% of all hernias [1]. However, over $3.4 billion in the United States is spent on ventral hernia repair for roughly 348,000 surgeries performed annually [1,2]. Ventral hernias are non-inguinal, non-hiatal defects in the abdominal wall fascia [2]. These abdominal defects can be congenital or acquired. Common causes of acquired ventral hernias include postoperative, incisional hernias, trauma, or repetitive stress, such as gaining or losing weight [3-5]. The European Hernia Society (EHS) classifies ventral hernias as primary or incisional. Primary hernias less than 2 cm are considered small, those more than 2 cm but less than 4 cm are considered medium sizes, and those 4 cm or more are considered large hernias [6]. Large ventral hernias can present a surgical challenge, especially when a loss of domain (LOD) is present [7]. Component separation is the primary technique for repairing large complex ventral hernias and represents the state of the art to date [3].

Multiple methods help achieve anatomical restoration after LOD in complex large ventral hernias. Commonly used techniques include open anterior component separation versus open posterior component separation and transverse abdominis muscle release [3,8]. A combination of a component separation technique and mesh utilization has lessened the recurrence rates of ventral hernias compared to no mesh reparation management [3,9,10]. Ventral hernia recurrence varied significantly by mesh type [10]. There are two main types of mesh, hybrid and nonhybrid. The hybrid mesh contains a permanent component intertwined with a hybrid component. In this case, with Tela Biologics (Malvern, PA, USA) hybrid mesh, the permanent synthetic material, monofilament polypropylene woven in a lock-stitch fashion, strengthens the hybrid layer of the bovine-derived extracellular membrane [10,11]. Further, the Phasix^TM^ (Becton Dickinson, Franklin Lakes, NJ, USA) absorbable nonhybrid synthetic mesh contains a monofilament resorbable mesh and a hydrogel barrier [12]. Nevertheless, the consensus on the advantages and disadvantages of different types of mesh and the optimal repair method with the best outcomes remains to be determined. In this complex large ventral hernia repair, we present a double mesh utilization, first, a Tela Biologics hybrid mesh placed underlay and then a Phasix^TM^ absorbable synthetic nonhybrid mesh placed onlay to reduce recurrence rates and complications and improve patient outcomes.

## Case presentation

We present a 50-year-old Caucasian male with a body mass index (BMI) of 27.4, one pack-day smoking history for an unknown number of years, and with presumed 20+ years. The patient denied ever being diagnosed with high blood pressure or diabetes. Further, the patient denied any alcohol or illicit drug consumption. The patient was involved in a traumatic motorcycle accident, resulting in a chronic diaphragmatic hernia rupture in February 2021. The patient had a large acute right diaphragmatic hernia rupture with herniation of the small bowel loops, colon, and liver that was repaired using a right thoracoabdominal approach and mesh. The patient was left with an open abdomen with an ABTHERA (3M, St. Paul, MN, USA) vacuum that was removed successfully with no further complications. After unsatisfied results from the first repair, the patient presented for plastic surgery consultation in October 2021.

**Figure 1:**
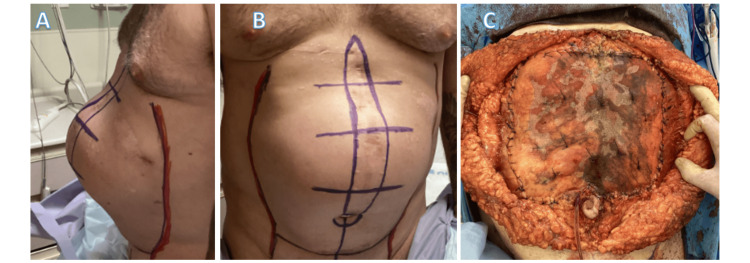
(A) Lateral and (B) anterior views of preprocedural large hernia; (C) underlay hybrid mesh as a bolster for posterior component separation of transversalis muscle.

At the time of the consultation, the patient presented with a >18 cm abdominal wall defect status post-traumatic diaphragmatic hernia repair (Figure 1A) and a left upper abdominal incisional hernia. A physical exam showed degenerative spondylosis and scoliosis on the bilateral hips, and sacroiliac joint and symphysis pubis degeneration were noted. No destructive bone lesions were seen. A chest X-ray showed no evidence of cardiomegaly or chronic interstitial lung changes. The patient was noted to have an elevated right hemidiaphragm, and mediastinal wires were seen in place. Bony structures appeared intact. The patient had a significant xiphoid umbilical hypertrophic scar on physical examination, which widened at least 3 cm at the supraumbilical zone. The patient also had a significant herniation in the epigastrium, umbilical, and ventral abdomen. Further, the rectus muscles had a greater than 5 cm widening and an abdominal keloid of 30 cm × 6 cm (Figures 1A, 1B). 

An extensive complex abdominal wall reconstruction with bilateral advanced fascial flaps component separation and repair of the abdominal wall defect was planned to restore the appropriate abdominal wall contour. Further, an abdominal computed tomography (CT) showed that within the hernia defect were loops of the colon, small bowel, and omentum with loss of abdominal domain. The surgical plan was modified after the imaging and consultation with the patient. A two-stage surgery was planned, with the first being a diagnostic laparoscopy with air insufflation followed by stage two-component separation surgery. The procedure was described to the patient, including risks, benefits, and alternatives. The patient was advised on smoking cessation for at least six weeks before the operation. The patient voiced understanding, agreed to the procedure, and signed the consent. The surgery was scheduled for December 2021; yet, the surgery was rescheduled due to staff testing positive for COVID-19. 

The first stage of the surgery was performed on January 11, 2022. Preoperative antibiotics were administered within 60 minutes of the first incision, standard skin preparation and draping were utilized, and all procedures were performed under general anesthesia. First, the diagnostic laparoscopy was performed to divide all intra-abdominal adhesions, freeing the adhesions from the abdominal wall and hernia sac to prevent potential iatrogenic injury. The pneumoperitoneum created for the laparoscopy helped test for diaphragmatic pressures as the patient had a significant diaphragmatic injury from prior trauma and had mesh repair of his right hemidiaphragm. Further, this pneumoperitoneum would stretch the tissues, enabling a more solid closure during the second stage-component separation.

The second stage of the surgery, component separation, was performed on March 10, 2022. The patient was a candidate for component separations surgery because the LOD was >18 cm. First, an incision about 5 to 10 mm outside the bounds of the keloid scar was made. The anterior sheath was dissected following a tumescent solution pathway down to good tissue. This dissection allowed us to raise significant fasciocutaneous flaps laterally and remove any Scarpa's fascia that remained laterally to the defect. The keloid scar was removed carefully utilizing loupe magnification, and preperitoneal fat bulging was found through the area under the scar in the midepigastric, umbilical, and ventral zones. The incision was extended, allowing access to the rectus musculature. An omentectomy was performed after removing the remaining abdominal adhesions and checking the area for any other blockages. The omentectomy was significant and allowed for easy replacement of the organs back into the abdominal cavity. 

Careful dissection delineated the right and left transversalis muscles, which served as a bolster zone where the biologic hybrid mesh was placed to secure the entire area (Figure 1C). The mesh was secured with parachute sutures of 0-Vicryl (Ethicon/J&J, Bridgewater, NJ) plus on each side, providing traction and stable placement of the submuscular mesh, creating a bridge. ACell powder was placed over the biologic hybrid mesh to aid in wound healing. Progressive tension sutures of 0-Vicryl were placed in a figure-of-eight manner over the transversalis fascia, bringing the rectus muscles overlying the bridge into position, which were reinforced by bringing the anterior sheath together in this manner, correcting the epigastric, umbilical, and ventral hernias. Then, fasciocutaneous flaps were undermined, any excess was trimmed, and irrigation was done with bacitracin and 1% betadine. Then, a large piece of Phasix^TM^ absorbable nonhybrid mesh was placed with 2-0 Vicryl plus sutures in an onlay fashion. This mesh added further support for the component separation. Three Jackson-Pratt (JP) drains were placed. The first drain was placed under the muscle overlying the bridge mesh and brought out through a lower midline stab incision. Two paramedian incisions were made to bring out the JP drains along each lateral gutter. The flaps were closed with 2-0 Neurolon and 2-0 PDS (Ethicon) in layers, while progressive tension sutures were placed with 2-0 PDS. 3-0 Prolene sutures were placed percutaneously throughout the wound to coapt the edges and secure the wound.

**Figure 2:**
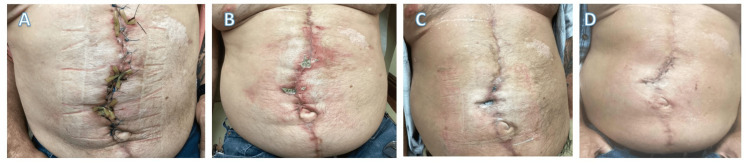
Anterior view of postprocedural hernia repair at (A) seven days postop, (B) 30 days postop, (C) 60 days postop, and (D) 90 days postop.

The patient had an intensive care unit (ICU) stay for over a week due to atelectasis and alcohol withdrawal. Alcohol dependence was not disclosed before surgery. He was safely discharged to a rehabilitation facility for seven days and then home with local wound care. He had a home nurse apply Silvadene, a Telfa dressing (Dublin, OH, USA), a xeroform pad, and tape to the wound daily. Seven days posthospital discharge, the patient had no obvious start of dehiscence (Figure 2A). The operating surgeon suggested weekly follow-ups. 

One-month follow-up revealed a slight dehiscence in the periumbilical area with visible granulation tissue (Figure 2B). The patient was heavily counseled on smoking cessation pre- and postoperation. He was also recommended to take plant-based zinc, vitamin C, and multivitamins twice daily. The second wound dehiscence occurred at the 90 days postoperative follow-up (Figure 2D). In both cases, 30% benzoyl peroxide was placed over the wound, and a weekly follow-up was scheduled with a corresponding improvement in healing. The patient was seen monthly for seven more follow-ups with no further complications. The patient was not compliant with smoking cessation. However, there were no more episodes of wound dehiscence in the following appointments. The last follow-up was at 10 months postoperative, in which the patient was again heavily counseled on smoking cessation and the risk that smoking presents for wound healing. The patient denied fever, chest pain, and shortness of breath. The wound did not show signs of dehiscence. The patient was referred to primary care for continued monitoring.

## Discussion

Our case details the use of both a hybrid (Tela Biologics) and synthetic absorbable (Phasix^TM^) mesh. To our knowledge, only two case series have been reported that use this technique; however, the types of mesh used in both studies differ from this case. The first repair by Azar et al. used an acellular matrix and soft synthetic or biologic mesh [13]. The second repair by Shaikh et al. used an acellular dermal collagen implant and a synthetic mesh [14]. The studies compared the safety and efficacy of the double underlay and onlay mesh, also known as the *sandwich* technique, on patients with LOD hernias and large defect ratios to those with smaller hernia defect ratios. Postoperative complications and recurrence rates were similar in both groups, around 10%. Both studies suggest this technique is safe and durable to restore the abdominal wall integrity with minimal recurrence rates. To date, our case reinforces these two published repairs.

Component separation is the most accepted procedure for large complex ventral hernias and usually requires using mesh [3,15]. Component separation can be performed with an anterior or posterior approach. In the open anterior approach (OAP) or external oblique technique, the objective is to release the external oblique from the internal oblique muscle to allow for medialization of the rectus muscle and closure of the defect [8,15]. To do so, large bilateral skin flaps are delineated, about 5 cm beyond the midline, to expose the lateral portions of the rectus [15]. The external oblique muscle is incised 2 cm lateral to the linea semilunaris and extended superiorly and inferiorly while separating it from the internal oblique muscle [8,15]. Then, the mesh is placed onlay to reinforce the closure. Even so, this technique is estimated to allow for tension-free closure of defects up to 10 cm in diameter; the subcutaneous dissection has been associated with seromas, wound infection, and abscess formation [16]. Further, the lateral abdominal area where the external oblique was divided can develop a lateral abdominal wall hernia; due to the nature of the division, there is no possibility of further anterior component separation division should the hernia recur [16].

On the other hand, the main goal of posterior component separation or transversus abdominis release (TAR) is to access the posterior rectus sheath by developing the retromuscular space from the medial rectus into the space between the transversus abdominis and internal oblique [15,16]. This technique allows for the placement of the mesh sublay above the posterior fascial layer and the internal oblique muscle [16,17]. The TAR technique is the best option for large hernias due to the preservation of the neurovascular bundles innervating the medial abdominal wall and its versatility and durability in abdominal wall reconstruction [8,15,16]. Further, a meta-analysis by Jones et al. demonstrated a hernia recurrence rate of 4.5% for the TAR technique versus 9.5% for the OAP [16]. In this case, we report using the TAR technique in which a hybrid mesh was placed underlay the rectus muscle with additional reinforcement of a synthetic non-hybrid mesh placed onlay. The addition of a mesh in abdominal wall reconstruction has been a topic that has been widely investigated, and results demonstrate that the recurrence rate is lower when used in the TAR approach when compared to an anterior technique [15,16]. 

The use of the double mesh in sizeable abdominal wall defects is still a relatively new technique amenable to different types of repair variations. However, compared to other surgical treatments, the *sandwich technique* with intraperitoneal mesh displayed the best results [18]. More recently, the mesh type (biologic, synthetic, or hybrid) has been a more controversial topic. Much evidence supports using mesh during hernia repairs, but limited investigations evaluate the properties of each mesh type [19]. Lake et al. evaluated the antimicrobial properties of hybrid and synthetic mesh. The results demonstrated that the synthetic absorbable mesh (Phasix^TM^) had no bacterial colonization, while four of the five-hybrid mesh did [19]. Much evidence supports using mesh during hernia repairs, but limited investigations evaluate the properties of each mesh type [19]. Further, the partially absorbable products provide a collagen network for tissue regeneration and reinforced strength [17]. As surgeons continue to shift toward decreasing the use of permanent prosthetics, a hybrid and synthetic absorbable mesh may be a good solution, as the recurrence rates seem lower [17]. However, future studies should focus on longer term follow-up to adequately report long-term outcomes. Positive and negative outcomes should be reported to complete the panorama on the benefits and risks of double mesh utilization in large ventral hernias repair. Furthermore, analysis of mesh cost should be a priority in this post-COVID-19 medical world.

## Conclusions

We report a case that used the double underlay and onlay mesh technique with a hybrid mesh under the muscle bridging the gap and progressive tension sutures placed on the fascia overlying the rectus, which was then reinforced with a synthetic nonhybrid absorbable mesh. Surgeons should know that using a biologic hybrid mesh and synthetic absorbable nonhybrid mesh could offer potential strength benefits to abdominal wall repair procedures and reduce the recurrence rates. Further, the choice of mesh affects the long-term postoperative outcomes in ventral hernia repair. When choosing a mesh, surgeons should consider factors such as the type of repair, the patient's premorbid conditions, and the patient's lifestyle.
